# Second-hand smoking, hypertension and cardiovascular risk: findings from Peru

**DOI:** 10.1186/s12872-021-02410-x

**Published:** 2021-12-03

**Authors:** Antonio Bernabe-Ortiz, Rodrigo M. Carrillo-Larco

**Affiliations:** 1 CRONICAS Center of Excellence in Chronic Diseases, Universidad Peruana Cayetano Heredia, Lima, Peru; 2 Universidad Científica del Sur, Lima, Peru; 3 Department of Epidemiology and Biostatistics, School of Public Health, Imperial College London, London, UK

**Keywords:** Second-hand smoking, Hypertension, Cardiovascular risk, Peru

## Abstract

**Background:**

Second-hand smoking has not been detailedly studied in Peru, where smoking is prohibited in all indoor workplaces, public places, and public transportation. Second-hand smoke exposure may occur at home or any other places. This study aimed to estimate the prevalence of second-hand smoking and assess its association with hypertension and cardiovascular risk in Peru.

**Materials and methods:**

Secondary analysis of a nationally-representative population-based survey including individuals aged 18–59 years. There were two outcomes: hypertension and 10-year cardiovascular risk using the Framingham and the 2019 World Health Organization (WHO) risk scores. The exposure was self-reported second-hand smoking during the 7 days before the survey. The association between second-hand smoking and hypertension was quantified with Poisson models reporting prevalence ratio (PR) and 95% confidence interval (95% CI); the association between second-hand smoking and cardiovascular risk was quantified with linear regressions reporting coefficients and their 95% CI.

**Results:**

Data from 897 individuals, mean age: 38.2 (SD: 11.8) years, and 499 (55.7%) females, were analyzed, with 8.7% subjects reporting second-hand smoking at home and 8.3% at work or any other place. Thus, 144 (15.5%; 95% CI: 12.8%-18.6%) subjects reported any second-hand smoking. In multivariable model second-hand smoking was associated with hypertension (PR = 2.42; 95% CI: 1.25–4.67), and with 1.2% higher Framingham cardiovascular risk, and 0.2% higher 2019 WHO risk score.

**Conclusions:**

There is an association between second-hand smoking and hypertension as well as with cardiovascular risk, and 15% of adults reported second-hand smoke exposure overall with half of them exposed at home. There is a need to guarantee smoking-free places to reduce cardiovascular risk.

## Introduction

Worldwide, the burden of cardiovascular disease (CVD) and hypertension is a public concern. From 1990 to 2017, the CVD death rate has decreased in high-income countries, but it remained the same in lower- and middle-income countries [1]. High systolic blood pressure, the leading risk factor for CVD, accounted for 10.8 million deaths globally (19·2% of all deaths) in 2019 [2]. In addition, the number of subjects aged 30–79 years with hypertension doubled from 1990 to 2019, despite stable global age-standardized prevalence [3]. In the Americas, the prevalence of raised blood pressure has decreased in all subregions; however, estimates showed a high level of heterogeneity between countries [4]. In Peru, for example, the age-standardized prevalence of hypertension increased from 18.7% in 2015 to 20.6% in 2018 [5].

Although active smoking is a well-known risk factor for hypertension and cardiovascular disease, and worldwide, 1.9 million people died from tobacco-induced heart disease [6], emerging studies have started to assess the association between second-hand smoking and cardiovascular outcomes. Thus, a recent systematic review using 10 cohort and 8 case–control studies, mainly from high-income countries, reported that second-hand smoking was associated with increased risk of CVD incidence and mortality [7]. A previous systematic analysis also found that the association between second-hand smoking increased the risk of CVD and all-cause mortality, but mainly in China, while the risk was only modest in the United States [8].

Although second-hand smoking has been detailed in high-income countries, this has been little studied in resource-constrained settings. In Peru, smoking is prohibited, by law [9], in all indoor workplaces, public places, and on public transportation. Nevertheless, passive smoking can occur at home or any other places, and for instance, estimations of the burden of second-hand smoking in resource-constrained settings are needed. Therefore, the main aim of this manuscript was to estimate the prevalence of second-hand smoking and assess its association with hypertension and cardiovascular risk using a nationally-representative survey in Peru.

## Methods

### Study design

This is a secondary analysis of a nationally-representative population-based survey named “The Food and Nutrition Surveillance Survey” (VIANEV, acronym in Spanish) and conducted in the period of 2017–2018 [10].

### Study population

Individuals aged 18 to 59 years, with a fasting period of at least 9 h but not more than 12 h were potentially eligible to participate. Pregnant women, adults taking a medication affecting glucose or lipid profile, those with gastrointestinal problems affecting usual diet, and those with a congenital condition or any problem preventing anthropometric assessment were excluded. For this assessment, records with complete data of second-hand smoking and hypertension and variables related to cardiovascular risk (i.e., systolic blood pressure, glucose, total cholesterol, etc.) were included.

### Sampling strategy

This survey selected individuals using a multistage random sampling approach and has been reported elsewhere [10]. Briefly, three different strata were used: urban, rural, and Metropolitan Lima/Callao. Clusters were selected using a random systematic procedure with a probability proportional to the adult population size, totaling 621 clusters (176 in Lima, 260 in urban settings, and 185 in rural areas), with the selection of six households in Lima and urban settings, and eight households in rural areas. Further, households were selected whether they have at least an individual in the proposed age range, and using a random and systematic technique. Eligible participants were invited to participate and an informed consent was applied. If a subject rejected participation, a replacement was selected using a random approach.

### Definition of variables

*Outcome* Two were the outcomes of interest. The first one was hypertension status, defined according to the Joint National Committee on Prevention, Detection, Evaluation, and Treatment of High Blood Pressure (JNC-7) [11]. Thus, a subject with hypertension had a systolic blood pressure (SBP) ≥ 140 mmHg, or a diastolic blood pressure (DBP) ≥ 90 mmHg, or reported having previous physician diagnosis of high blood pressure levels.

The second outcome was cardiovascular risk, evaluated using the Framingham 10-year cardiovascular disease event risk prediction [12] and the World Health Organization (WHO) 10-year risk of cardiovascular disease (CVD) [13]. The decision to use two different cardiovascular risk scores was based on the poor concordance between current risk scores showing the uncertainty of selecting any of them for public health and clinical interventions in Peru [14]. The Framingham score utilizes the sex, age (in years), systolic blood pressure (mmHg), use of anti-hypertensive medication, smoking status, diabetes status, high-density lipoprotein level (mg/dL), and total cholesterol (mg/dL). On the other hand, the WHO risk score used data from the Emerging Risk Factors Collaboration and recalibrates the predicted risk scores to reflect the expected 10-year risk in contemporary populations in 21 global regions, using the sex, age (in years), smoking status, history of diabetes, systolic blood pressure (mmHg), total cholesterol (mmol/L), and body mass index (kg/m^2^). Both risk scores were included as a percentage numerical value for analysis.

*Exposure* Second-hand smoking was the exposure in this analysis, and defined if the subject reported passive smoking at home or at work [15]. This assessment was based on the same questions as a previous worldwide report [16]: “During the last 7 days, on how many days did a member of your household smoke in your presence?” and “During the past 7 days, on how many days did someone smoke indoors at work (office, building) and in your presence? For descriptive purposes, both variables were analyzed separately; but, for main analysis, both were combined and total second-hand smoking was considered whether the participant reported to be exposed to passive smoking for at least 1 day [15]. Unfortunately, data about degree or duration of exposure were not collected in the survey, and for instance, was not available for analyses.

*Co-variables* Other variables were utilized in the analysis as potential confounders. Sociodemographic variables included sex (female vs. male), age (< 30, 30–39, 40–49, and 50–59 years), education level (< 7 years, 7–11 years, and 12 + years), and socioeconomic position, built based on household assets, and split into tertiles (low, middle, and high). In addition, behavioral variables were also added, including current smoking (no current smoking, current smoking, and daily smoking), alcohol drinking (no in the last 12 months, in the last 12 months but not in the last 30 days, and in the last 30 days), and physical activity, using the short version of the International Physical Activity Questionnaire (moderate/high vs. low levels).

### Statistical analysis

STATA 16 for Windows (StataCorp, College Station, TX, US) was used for analysis. All the analysis were performed using appropriate statistical techniques for complex sample and subpopulation analyses [17]. An initial description of the study population was conducted using mean and standard deviation (SD) for numerical variables, and frequencies and proportions for categorical ones.

Prevalence and 95% confidence intervals (95% CI) were pursued for our exposure and hypertension outcome, whereas mean and 95% CI was used for estimating overall 10-year cardiovascular risk. Comparisons were conducted using the Rao-Scott Chi-squared test for categorical variables, and the F-test for numerical ones.

To assess the association between second-hand smoking and hypertension, crude and adjusted Poisson regression models were used, reporting prevalence ratios (PR) and 95% CI. On the other hand, crude and adjusted linear regression models were utilized to assess the association between second-hand smoking and 10-year cardiovascular risk, reporting coefficients and 95% CI.

## Results

### Description of the study population and second-hand smoking

A total of 1093 subjects were enrolled in the study, but 196 (17.9%) records were excluded from analysis: 149 with no complete information, and 47 because pregnancy, puerperium or breastfeeding. Thus, information from 897 individuals, mean age: 38.2 (SD: 11.8) years, and 499 (55.7%) females, was analyzed.

Seventy-seven (8.7%; 95% CI: 6.6–11.3%) subjects of the total sample reported second-hand smoking at home on 1 or more days, 2.4% (95% CI: 1.4–4.0%) on 3 or more days, and 1.3% (0.7–2.6%) daily during the past 7 days. Similarly, 77 (8.3%; 95% CI: 6.4–10.6%) individuals reported second-hand smoking at work or any other place different from home on 1 or more days, 2.0% (95% CI: 1.1–3.7%) on 3 or more days, and 0.5% (95% CI: 0.2–1.8%) daily during the past 7 days. Thus, 144 (15.5%; 95% CI: 12.8–18.6%) subjects reported second-hand smoking exposure in any place. Total second-hand smoking was associated with age (p = 0.002), education level (p = 0.003), current smoking status (p < 0.001), and alcohol drinking (p < 0.001). See details in Table 1.

**Table 1:** Characteristics of the study population by total second-hand smoking status

	Total second-hand smoking		p-value*
	No (n = 753)	Yes (n = 144)	
*Sex*			0.08
Female	432 (57.3%)	67 (47.1%)	
Male	321 (42.7%)	77 (52.9%)	
*Age*			0.002
< 30 years	188 (26.3%)	59 (43.6%)	
30–39 years	196 (25.3%)	29 (21.1%)	
40–49 years	195 (26.0%)	29 (15.6%)	
50–59 years	174 (22.4%)	27 (19.7%)	
*Education level*			0.003
< 7 years	193 (20.0%)	17 (6.8%)	
7–11 years	278 (35.3%)	63 (43.4%)	
12 + years	282 (44.7%)	64 (49.7%)	
*Socioeconomic position*			0.45
Low	258 (29.6%)	42 (26.3%)	
Middle	251 (32.0%)	41 (28.9%)	
High	244 (38.4%)	61 (44.9%)	
*Current smoking*			< 0.001
No current smoking	682 (89.0%)	98 (67.5%)	
Current smoking	59 (9.2%)	36 (23.2%)	
Daily smoking	12 (1.8%)	10 (9.3%)	
*Alcohol drinking*			< 0.001
No in the last 12 months	203 (23.0%)	19 (10.6%)	
Last 12 months, but not last 30 days	213 (27.6%)	24 (17.2%)	
During last 30 days	337 (49.4%)	101 (72.2%)	
*Physical activity*			0.74
Moderate/high levels	545 (70.7%)	104 (72.2%)	
Low levels	208 (29.3%)	40 (27.8%)	

*Comparisons were conducted taking into account the multistage sampling design using Rao-Scott Chi square test

### Hypertension and cardiovascular risk

The prevalence of hypertension was estimated in 10.0% (7.9–12.5%) and although it was more common among men (12.0%) compared to women (8.2%), this difference was not significant. Hypertension status was associated with age (p < 0.001) and education level (p = 0.03) as shown in Table 2.

**Table 2:** Characteristics of the study population by hypertension status

	Hypertension status		p-value*
	No (n = 799)	Yes (n = 98)	
*Sex*			0.06
Female	447 (56.8%)	52 (46.3%)	
Male	352 (43.2%)	46 (53.7%)	
*Age*			< 0.001
< 30 years	236 (30.8%)	11 (12.7%)	
30–39 years	211 (26.0%)	14 (12.9%)	
40–49 years	201 (24.4%)	23 (23.8%)	
50–59 years	151 (18.8%)	50 (50.7%)	
*Education level*			0.03
< 7 years	178 (16.7%)	32 (29.0%)	
7–11 years	308 (36.9%)	33 (33.7%)	
12 + years	313 (46.4%)	33 (37.3%)	
*Socioeconomic position*			0.79
Low	270 (29.3%)	30 (26.9%)	
Middle	261 (31.7%)	31 (29.8%)	
High	268 (39.0%)	37 (43.3%)	
*Current smoking*			0.33
No current smoking	693 (85.5%)	87 (87.0%)	
Current smoking	85 (11.3%)	10 (12.4%)	
Daily smoking	21 (3.2%)	1 (0.6%)	
*Alcohol drinking*			0.52
No in the last 12 months	197 (20.8%)	25 (23.7%)	
Last 12 months, but not last 30 days	207 (25.6%)	30 (29.4%)	
During last 30 days	395 (53.6%)	43 (46.9%)	
*Physical activity*			0.62
Moderate/high levels	579 (71.2%)	70 (68.1%)	
Low levels	220 (28.8%)	28 (31.9%)	

*Comparisons were conducted taking into account the multistage sampling design using Rao-Scott Chi square test

Using the Framingham score, 10-year cardiovascular risk was, on average, 4.9% (95% CI: 4.4–5.5%), and 14.2% (95% CI: 11.9–16.9%) had a risk ≥ 10%. Score of the Framingham cardiovascular risk was greater among men (p < 0.001), older individuals (p < 0.001), among those with high socioeconomic position (p < 0.001), and those with smoking habits (p < 0.001). Details are shown in Table 3.

**Table 3:** Characteristics of the study population by cardiovascular risk

	Cardiovascular risk			
	Framingham risk score		WHO cardiovascular risk score	
	Mean (95% CI)	p-value	Mean (95% CI)	p-value
*Sex*		< 0.001		< 0.001
Female	2.9% (2.5–3.3%)		1.3% (1.2–1.5%)	
Male	7.5% (6.5–8.5%)		2.0% (1.8–2.2%)	
*Age*		< 0.001		< 0.001
< 30 years	0.9% (0.6–1.0%)		0.5% (0.4–0.6%)	
30–39 years	2.9% (2.4–3.5%)		1.0% (0.8–1.1%)	
40–49 years	5.3% (4.6–6.0%)		1.7% (1.5–1.9%)	
50–59 years	12.3% (10.5–14.1%)		3.7% (3.2–4.1%)	
*Education level*		0.80		0.33
< 7 years	5.2% (4.3–6.0%)		1.8% (1.5–2.0%)	
7–11 years	5.1% (4.2–5.9%)		1.6% (1.4–1.8%)	
12 + years	4.8% (3.9–5.6%)		1.6% (1.3–1.8%)	
*Socioeconomic position*		< 0.001		< 0.001
Low	3.6% (3.0–4.2%)		1.3% (1.1–1.5%)	
Middle	4.8% (3.9–5.8%)		1.6% (1.3–1.8%)	
High	6.0% (5.0–7.1%)		1.9% (1.6–2.1%)	
*Current smoking*		< 0.001		< 0.001
No current smoking	4.2% (3.8–4.7%)		1.4% (1.3–1.5%)	
Current smoking	8.9% (6.1–11.7%)		2.8% (2.1–3.5%)	
Daily smoking	10.8% (5.9–15.7%)		3.6% (2.3–4.8%)	
*Alcohol drinking*		0.13		0.09
No in the last 12 months	4.4% (3.5–5.3%)		1.5% (1.2–1.7%)	
Last 12 months, but not last 30 days	4.4% (3.5–5.4%)		1.4% (1.2–1.6%)	
During last 30 days	5.4% (4.6–6.3%)		1.7% (1.5–1.9%)	
*Physical activity*		0.98		0.96
Moderate/high levels	4.9% (4.3–5.6%)		1.6% (1.4–1.8%)	
Low levels	5.0% (4.0–6.0%)		1.6% (1.3–1.9%)	

*Comparisons were conducted taking into account the multistage sampling design

Nevertheless, when using the WHO cardiovascular risk score, the 10-year risk was, on average, 1.6% (95% CI: 1.5–1.8%), and 0.5% (95% CI: 0.1–1.8%) had a risk ≥ 10%. Similar to the Framingham score, the WHO risk score result was greater among men (p < 0.001), older individuals (p < 0.001), among those with high socioeconomic position (p < 0.001), and those with smoking habits (p < 0.001). See Table 3.

### Second-hand smoking and cardiovascular risk

In multivariable model, and controlling for sex, age, education level, socioeconomic position, current smoking status, alcohol drinking and physical activity, total second-hand smoking was associated with greater probability of hypertension (PR = 2.42; 95% CI: 1.25–4.67). In addition, second-hand smoking was also associated with an increase of 1.2% in the Framingham cardiovascular risk, whereas this increment was 0.2% in the WHO risk score (Table 4).

**Table 4:** Association between second-hand smoking and cardiovascular risk: crude and adjusted models

	Prevalence	Crude model	Adjusted model*
	n/N (%)	PR (95% CI)	PR (95% CI)
*Hypertension*			
Total second-hand smoking			
No	90/880 (9.1%)	Reference	Reference
Yes	21/151 (14.5%)	1.70 (0.92–3.13)	2.42 (1.25–4.67)

Cardiovascular risk (10-year)	Mean	Crude model	Adjusted model*
	Mean (95% CI)	β (95% CI)	β (95% CI)
*Framingham risk score*			
Total second-hand smoking			
No	4.7% (4.2–5.3%)	Reference	Reference
Yes	6.2% (4.7–7.8%)	1.5% (0.0–3.1%)	1.2% (0.1–2.3%)
*WHO cardiovascular risk score*			
Total second-hand smoking			
No	1.6% (1.4–1.7%)	Reference	Reference
Yes	1.9% (1.5–2.3%)	0.3% (0.0–0.7%)	0.2% (0.1–0.5%)

Estimations were obtained taking into account the multistage sampling design

*Model adjusted for sex, age, education level, socioeconomic position, current smoking status, alcohol drinking, and physical activity

## Discussion

### Main findings

Our analyses evidence that, after controlling for several confounders, there is an association between second-hand smoking and hypertension and 10-year cardiovascular risk assessed by the Framingham and the WHO risk scores. Of relevance, despite of the Peruvian law prohibiting smoking in all indoor and public places, 15% of adults aged 18 and 59 years reported second-hand smoke exposure overall, and about 10% of subjects are exposed at home.

### Comparison with previous studies

A systematic review demonstrated the association of second-hand smoking and cardiovascular disease by including information of 18 studies mainly for high income countries [7]. This study showed an increased between 12% in the risk of CVD in 10 cohort studies compared to 28% risk increase in 8 case–control studies. In addition to that, two population-based studies, conducted in never smokers has been associated with cardiovascular conditions. Thus, a cohort study reported an important association between passive smoking and coronary artery calcification [18], with a remarkable dose–response: 54% increase among low second-hand smoking exposure, 60% for those with moderate exposure, and 93% risk increase in those with high exposure. On the other hand, in Korean subjects, a study found an association between second-hand smoke exposure and hypertension [19]. This association, however, was present only among women than men, with an increase of 50% in the prevalence of hypertension among those with a exposure ≥ 2 h/day compared to no exposure. In a study conducted in China enrolling over five million females aged 20 to 49 years, an association between husband smoking, including amount and cumulative exposure, with female hypertension prevalence was reported [20]; thus, the probability of having hypertension among wives increased from 22 to 75% when the husband smoked from 1-to-5 and ≥ 21 cigarettes per day, respectively. Finally, a study in Japan including 32,098 study subjects reported an increment of 3% in the probability of having hypertension per each hour per day increase in second-hand smoke exposure, and was especially higher among men than women [21].

Although active smoking is used as an important predictor of cardiovascular risk, little emphasis has been put on second-hand smoking, especially in resource-constrained settings. Thus, the INTERHEART modifiable risk score is one of the few risk scores including second-hand smoking as one of the predictors of cardiovascular disease [22]. Therefore, our results are in the same line with previous reports, but expand on assessing 10-year cardiovascular risk in a Latin American country.

Finally, our estimates regarding second-hand smoking are below to those reported by other studies, even including those with previous smoking history. A global report documented that 40% of children, 33% of male non-smokers and 35% of female non-smokers were exposed to second-hand smoke [23]. In Canada, for example, about 25% of never-smokers and 30% of ex-smokers self-reported second-hand smoke exposure [24], whereas this estimate can be as high as 48% among non-smokers in different counties in China [25].

### Public health relevance

Second-hand smoking, similar to active smoking, has been associated with platelet activation, and for instance thrombus and plaque formation [26]. Endothelial lesions may appear due to vasoconstriction and the influx of inflammatory cells to the vascular wall. In addition, second-hand smoking is involved in the stiffening of arterial walls and, for instance, in the increase of arterial blood pressure [27].

Second-hand smoke exposure is a significant concern in different countries, and defining the problem and epidemiology of this risk factor seems to be relevant. Active smoking among adults is considered low in Peru, based not only in self-report but also using urine cotinine [28], and as a result, very few studies have been conducted to estimate the prevalence of second-hand smoking. In our study, of the total subjects reporting second-hand smoking, almost half of them reported exposure at home, which highlight the need of a family-based smoking restriction strategy to reduce the consequences of this exposure. This can be relevant as a recent study reported an estimate of 33% as the prevalence of second-hand smoke exposure among adolescents (i.e., those aged between 12 and 16 years) [16], which can be at high risk of future chronic conditions.

In addition to that, and despite of the smoking law in Peru, a relatively important proportion of subjects reported exposure at work or any other public place. Therefore, a better control and accomplishment of the law may be required. The previous experience in Europe established that a fully comprehensive smoke-free legislation is more effective than partial laws in reducing exposure to second-hand smoking [29]. Any law should be also actively enforced in order to have the desired impact. Thus, second-hand smoking could be used for assessing smoking-free efforts.

### Strengths and limitations

Strengths of this study include the nationally-representative and multistage approach as well as the assessment of second-hand smoking at home and at other places separately. However, this report also present limitations that should be highlighted. First, the cross-sectional nature prevents us to talk about causation, and only association may be inferred. Despite of this, our estimates are similar to those from previous literature. Second, passive smoking was assessed by self-report based on the 7 days previous to the survey, and for instance, we assumed a long second-hand smoking exposure when could not be the case inducing misclassification bias. We used this definition because of the questionnaire used in the survey we analyzed, which is consistent with other health surveys and other publications [16]. Future work should study whether other definitions change the prevalence estimates and correlates of second-hand smoking. Third, some recall bias may arise as part of self-reporting information. However, the impact on our estimates may be negligible as data was obtained on the 7 days previous to the survey. Fourth, degree or duration of exposure to second-hand smoking was not collected and, for instance, dose–response could not be explored. This is a shared limitation with other reports in the field looking national prevalence estimates. Finally, some residual confounding may arise as some confounders were not considered as health status and pre-existing chronic conditions.

## Conclusions

There is a positive association between second-hand smoking and hypertension as well as with cardiovascular risk, and 15% of adults reported second-hand smoke exposure overall, with a great number of them exposed at home. There is a need to enforce the Peruvian law to have smoking-free places as well as to include second-hand smoking as a potential way for smoking surveillance.

## Data Availability

The dataset used for this manuscript is freely available from the Centro Nacional de Alimentación y Nutrición (CENAN), part of the Instituto Nacional de Salud, under reasonable request.
